# Contributions of Central Command and Muscle Feedback to Sympathetic Nerve Activity in Contracting Human Skeletal Muscle

**DOI:** 10.3389/fphys.2016.00163

**Published:** 2016-05-13

**Authors:** Daniel Boulton, Chloe E. Taylor, Vaughan G. Macefield, Simon Green

**Affiliations:** ^1^School of Science and Health, Western Sydney UniversitySydney NSW, Australia; ^2^School of Medicine, Western Sydney UniversitySydney NSW, Australia; ^3^Neuroscience Research AustraliaSydney NSW, Australia

**Keywords:** cardiovascular control, muscle contraction, sympathetic, voluntary, electrical stimulation

## Abstract

During voluntary contractions, muscle sympathetic nerve activity (MSNA) to contracting muscles increases in proportion to force but the underlying mechanisms are not clear. To shed light on these mechanisms, particularly the influences of central command and muscle afferent feedback, the present study tested the hypothesis that MSNA is greater during voluntary compared with electrically-evoked contractions. Seven male subjects performed a series of 1-min isometric dorsiflexion contractions (left leg) separated by 2-min rest periods, alternating between voluntary and electrically-evoked contractions at similar forces (5–10% of maximum). MSNA was recorded continuously (microneurography) from the left peroneal nerve and quantified from cardiac-synchronized, negative-going spikes in the neurogram. Compared with pre-contraction values, MSNA increased by 51 ± 34% (*P* < 0.01) during voluntary contractions but did not change significantly during electrically-evoked contractions (−8 ± 12%, *P* > 0.05). MSNA analyzed at 15-s intervals revealed that this effect of voluntary contraction appeared 15–30 s after contraction onset (*P* < 0.01), remained elevated until the end of contraction, and disappeared within 15 s after contraction. These findings suggest that central command, and not feedback from contracting muscle, is the primary mechanism responsible for the increase in MSNA to contracting muscle. The time-course of MSNA suggests that there is a longer delay in the onset of this effect compared with its cessation after contraction.

## Introduction

The control of sympathetic nerve activity to contracting skeletal muscle is important for the control of muscle perfusion and regulation of arterial blood pressure during movement and exercise. Noradrenaline release from sympathetic neurons constrains vasodilation and hyperaemia (Haug and Segal, 2005), and sympathetic constraint of muscle hyperaemia is important for blood pressure regulation when assuming an upright stance (Sprangers et al., 1991) and during physical activity across a wide range of intensities (Savard et al., 1987; Masuki and Nose, 2003). Given the complexity of vascular control (Segal, 2005; Saltin, 2007) and that perfusion in human muscles can be varied across a wide range and in proportion to muscle activity (Andersen and Saltin, 1985; Saunders et al., 2005; Reeder and Green, 2012), a mechanism linking the level of sympathetic constraint to intensity of muscle activity might be important for this local vascular control and system-wide regulation of arterial blood pressure (Boulton et al., 2014). Yet, evidence pertaining to the effect of contraction on muscle sympathetic nerve activity (MSNA) to active muscle is conflicting.

Three studies from different laboratories tested the effect of sustained (1–3 min), isometric contractions of leg muscles on sympathetic nerve activity to these muscles. Wallin et al. (1992) made bilateral MSNA recordings (peroneal nerve) during mild to moderate voluntary contractions (5–30%MVC) of the dorsiflexors in one limb and reported that MSNA to the active limb declined to levels lower than observed in the inactive limb. Hansen et al. (1994) recorded MSNA to toe extensor muscles during moderate voluntary contractions (20%MVC) and reported no effect of contraction on MSNA. Recently, we measured MSNA to the contracting *tibialis anterior* muscle during voluntary contractions (10–50%MVC) and observed an intensity-dependent increase in MSNA (Boulton et al., 2014). Thus, the evidence pertaining to the effect of contraction on MSNA to contracting muscle differs considerably between studies and, although the reasons are not clear, might relate somewhat to the different analytical approaches used.

Although the effect of contraction on MSNA is unclear, observations of an intensity-dependent increase in MSNA from our study (Boulton et al., 2014) are consistent with evidence of increased sympathetic outflow in contracting muscle during moderate-to-high intensity dynamic contractions using a technique (noradrenaline spillover) *not* based on neural recordings (Savard et al., 1987). This increases the likelihood that contraction increases MSNA to contracting muscle and raises the possibility of a mechanism linking MSNA to the level of muscle activity. A contraction-dependent increase in MSNA to active muscle could be due to increased central command, upward resetting of the baroreflex, and/or increased muscle afferent feedback. Central command does not appear to increase MSNA to inactive muscles (Victor et al., 1989). By contrast, evidence pertaining to a rapid, positive effect of central command on sympathetic outflow to the coronary vascular bed (Matsukawa, 2012) raises the possibility that central command increases MSNA to active skeletal muscle. Muscle afferent feedback linked to mechanical and chemical stimuli—the muscle mechanoreflex and metaboreflex, respectively—contributes to the “exercise pressor reflex” and the increase in MSNA to *inactive* muscle (Murphy et al., 2011). Whether or not this feedback increases MSNA to *active* muscle is not clear. The rapid rise and fall in MSNA during and after contractions (Boulton et al., 2014) suggests involvement of a rapidly-acting mechanism, such as mechanical feedback (Cui et al., 2006) or central command. That MSNA falls rapidly after contraction despite the persistence of the metaboreflex (Boulton et al., 2014) suggests that metabolic stimuli do not contribute to this mechanism.

To shed light on this mechanism, we recorded MSNA to contracting muscle during and after sustained voluntary and electrically-evoked contractions. Theoretically, an increase in MSNA during voluntary contractions might be due to central command and/or muscle afferent feedback, whereas an increase in MSNA in response to direct muscle stimulation should be due to feedback from contracting muscle only. Assuming that central command and the mechanoreflex contribute independently to the rise in MSNA, we tested the hypothesis that the contraction-induced increase in MSNA is greater during voluntary than electrically-evoked contractions. In addition, we assessed the time-course of MSNA during and after contractions to provide additional insight into the mechanisms involved in its control. By performing weak static voluntary contractions and limiting contractions to 1 min, we minimized any potential contribution of metaboreceptors to the sympathetic response.

## Materials and methods

### Subjects and ethics

Experiments were performed on 13 normotensive male subjects aged 18–48 years. All experiments were conducted in accordance with the Declaration of Helsinki (2008) and approved by the Human Research Ethics Committee of Western Sydney University. All subjects provided written, informed consent prior to participation.

### Experimental design and protocol

MSNA was recorded continuously during a series of voluntary (V) and electrically-stimulated (ES) contractions involving dorsiflexor muscles of the left leg and performed in the supine position. As a robust test of the experimental hypothesis, we recorded MSNA (left leg) during and after a minimum of three contractions and up to a maximum of 10 contractions in each condition. For technical reasons, this minimum requirement could not be achieved in six subjects and, consequently, only recordings from the remaining seven subjects were used to test the hypothesis. The first and last contractions in the series were preceded and followed by 5 min rest periods, respectively. Each contraction was isometric and 1-min long, separated by 2 min of rest, and V and ES contractions were alternated throughout the protocol. In addition, the type of contraction performed first was counterbalanced between subjects. To minimize pain and the pressor response during electrical stimulation (see Discussion), the intensity of contractions was restricted to 5–10% of the highest force during two maximum voluntary efforts (MVC) performed immediately before insertion of needles into the nerve and muscle. To ensure similar forces during V and E contractions, the force of a voluntary contraction was matched to the force produced during the preceding ES contraction. Subjects were instructed to gradually increase and decrease the force at the beginning and end of the voluntary contractions over 4–5 s to minimize movement of the intraneural microelectrode.

### Recording procedures

Subjects laid supine on a custom-built ergometer (Green et al., 2011) with the left foot strapped in a plantarflexed position (~95°) to a footplate connected to a force transducer. The right leg and foot were placed in a comfortable position where they would not interfere with measurements from the left leg. The left common peroneal nerve was located at the fibular head using a 2 mm diameter probe that delivered constant-current stimuli (0.2 ms pulses, 2–10 mA) at 1 Hz (Stimulus Isolator, ADInstruments, Sydney, Australia). A tungsten microelectrode (Frederick Haer and Co, Bowdoin, ME, USA) was inserted at the optimal site for evoking muscle twitches and advanced toward the nerve while delivering weak electrical stimuli (0.02–1 mA) through the microelectrode; an adjacent uninsulated microelectrode served as the reference. The generation of twitches in the pretibial flexors, without radiating paraesthesia, at <20 μA indicated that the microelectrode was located in a muscle fascicle. Neural activity was amplified (gain 2 × 10^4^) and filtered (bandpass 0.3–5.0 kHz) using an isolated amplifier and headstage (NeuroAmpEX, ADInstruments, Sydney, Australia), and stored on computer (10 kHz sampling) using a computer-based data acquisition and analysis system (PowerLab 16SP hardware and LabChart 8 software; ADInstruments, Sydney, Australia). The location of the microelectrode in a muscle fascicle was confirmed by the presence of spontaneous or stretch-evoked activity of muscle spindle afferents, an absence of afferent response to stroking the skin, and the presence of spontaneous, cardiac-synchronized bursts of MSNA. The location of the microelectrode tip was adjusted to maximize the signal to noise ratio of MSNA such that discrete negative-going spikes of MSNA could be detected in an oligounitary recording.

A single lead (II) electrocardiogram (0.3–1 kHz) was recorded with Ag-AgCl surface electrodes (BioAmp, PowerLab, ADInstruments, Sydney, Australia) on the chest and sampled at 2 kHz. Respiration was recorded using a strain-gauge transducer (DC-100 Hz; Pneumotrace II, UFI, Morro Bay, CA, USA) around the chest and sampled at 100 Hz. Blood pressure was measured at the finger using continuous, non-invasive, beat-to-beat digital arterial plethysmography (Finometer Pro, Finapres Medical Systems, The Netherlands), which was height corrected for the difference between the finger and heart and sampled at 400 Hz.

### Stimulation procedures

Preparation for electrical stimulation of the left *tibialis anterior* muscle involved identifying its motor point by evoking the strongest twitch response from an external electrical stimulus. A low-impedance (<50 kΩ) insulated tungsten microelectrode was inserted at the motor point of this muscle to a depth of ~1 cm and attached to the cathode of a stimulator (Digitimer DS7A, Digitimer Ltd, UK) with the anode (Ag-AgCl surface electrode) placed on the lower half of the muscle. The stimulus pulse width was constant (100 ms) but the stimulus current (7–24 mA) and frequency (25–60 Hz) were varied until a contraction of ~10% MVC could be sustained for 1 min. EMG (10 Hz–1 kHz) was recorded using Ag-AgCl surface electrodes over the tibialis anterior of the contracting leg, sampled at 2 kHz and normalized to the EMG recorded during maximal voluntary contraction (MVC). Dorsiflexion force was measured using a load cell attached to the footplate (SUP 6 Tension Sensor, Altec Electronics, Shenzhen, China), amplified (gain 200x, bandpass DC-10 Hz; Quad Bridge Amplifier, ADInstruments, Sydney, Australia), sampled at 100 Hz and normalized to the MVC for each subject.

### MSNA analysis

**Figure 1:**
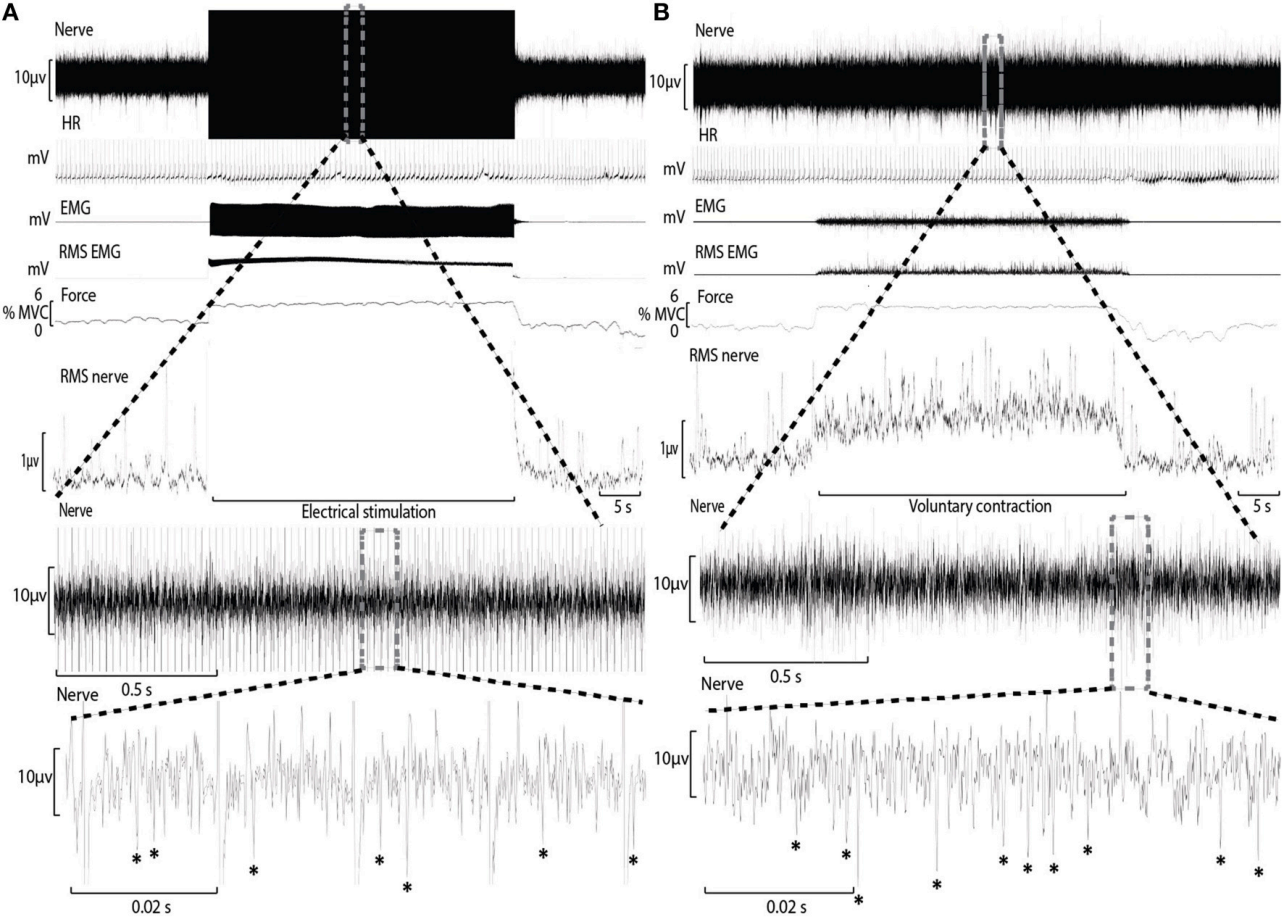
**Experimental records from one subject during electrical stimulation of tibialis anterior (A) and during a weak voluntary contraction at a matched intensity (B)**. A section of the “Nerve” channel during electrical stimulation has been expanded to reveal the raw neural signal between electrical artifact and sympathetic spikes have been identified with an asterisk (^*^). The “Nerve” channel during voluntary contraction has also been expanded for comparison.

Conventional analysis of MSNA is based on visual display of the root-mean-square of the neurogram, but display and identification of sympathetic bursts is corrupted by artifact during electrical stimulation (Figure 1). A potentially more sensitive approach of quantifying MSNA, not yet applied to recordings from contracting muscle, involves counting negative spikes of sympathetic origin that can be differentiated from spikes of non-sympathetic origin which include positive spikes related to motor efferent and afferent activity (Bent et al., 2006).

**Figure 2:**
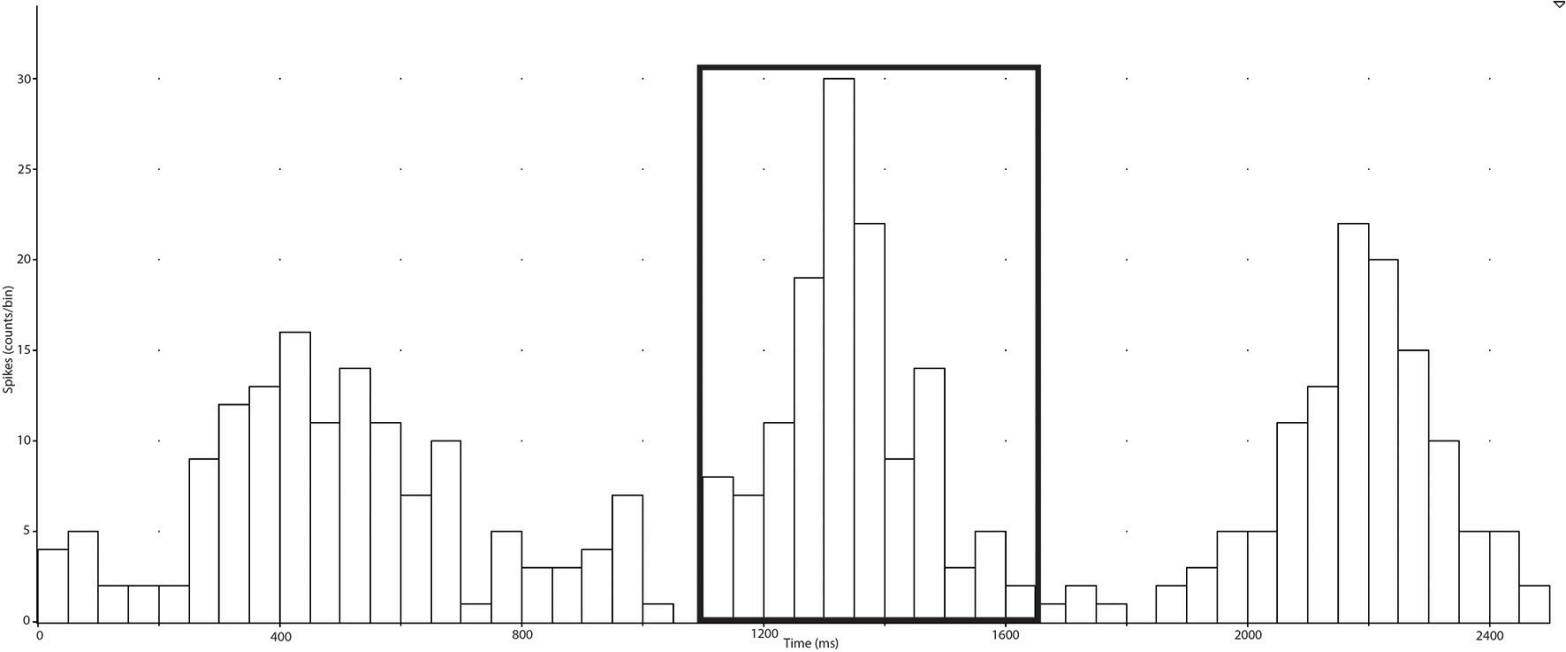
**Post-stimulus time histograms were used to measure the timing of neural spikes relative to cardiac beats (R-R intervals) of the selected period (collected in the spike train)**. MSNA, which is cardiac-locked, can be measured ~1200 ms after an R-R interval and the elevation of counts around this time is selected and the sum of the counts is recorded as the number of spikes during the selected period.

In the present study, negative spikes (half width 0.2–0.6 ms) were clearly observed in neurograms during contractions and detected using window discriminator software (Spike Histogram, LabChart 2.5, ADInstruments). To account for the delay between the R-wave (ECG) and MSNA in the peroneal nerve (Fagius and Wallin, 1980), the neurogram was shifted back in time (~1.15–1.30 s) relative to the R-wave. Autocorrelation histograms for the cardiac signal, as well as cross-correlation and post-stimulus time histograms between negative spikes and R-R intervals, were generated in 50 ms bins (Figure 2). Discriminator levels used to detect spikes were adjusted until spikes exhibited robust cardiac modulation in cross-correlations between spike counts and R-R intervals. Contractions with no evidence of robust cardiac modulation of MSNA were not analyzed and the rest and recovery periods associated with this contraction were also excluded. For each subject, an equal number of cardiac cycles were used to construct cross-correlation and post-stimulus time histograms pertaining to MSNA during contraction and rest periods. This was facilitated by the minimal effect of contraction on heart rate (see Results) and meant that any differences in spike counts between contraction and rest were not a function of heart rate and could be attributed to changes in MSNA burst intensity or incidence. MSNA spike counts were based on analysis of the number of spikes during 600 ms periods after each R-wave (i.e., diastole) and which centered about a peak spike count (Figure 2).

During ES, electrical artifact at the frequency of electrical stimulation obscured a small proportion of the recording period (Figure 1). Each recording of the neurogram during bouts of ES were analyzed for the proportion of time obscured by this artifact (mean ± SD = 7.9 ± 4.2%) and used to adjust the estimate of MSNA (counts^.^min^−1^) accordingly.

As a test of the validity of spike frequency estimates to assess changes in MSNA from rest to contraction, we compared the effects of voluntary contraction on MSNA spike frequency and MSNA estimated using a more conventional analytical technique based on the root-mean-square of the neurogram and identification of bursts of sympathetic activity. With this conventional technique we assessed the MSNA burst frequency, amplitude and their product (i.e., total MSNA) during rest and voluntary contraction (Boulton et al., 2014).

### Statistical analysis

The hypothesis predicts that the change in MSNA from rest to contraction is greater during V than ES. A two-way repeated measures ANOVA (IBM SPSS Statistics v.22, Chicago, IL) applied to MSNA (1-min average) was used to test for this interaction, as well as testing for main effects and interactions for other variables and exploring the time-course of MSNA during and after contraction. The level of significance was set at *p* ≤ 0.05 and results are expressed as mean ± SD.

## Results

### Rest and stability of MSNA

Neurograms and recordings of negative-going spikes during ES and V contractions are shown in Figure 1. The total number of spikes within a cardiac-locked burst was measured from the post-stimulus time histograms (Figure 2) for all cardiac cycles within a specified measurement period (15 or 60 s) and used to quantify MSNA in spikes per minute. MSNA averaged over 5 min during the initial and final rest periods of the protocol was not different (32.9 ± 2.9 vs. 31.3 ± 3.0 spikes/min; *P* = 0.41). MSNA during the final minute of rest before V and ES contractions was 32.5 ± 5.9 and 34.0 ± 7.9 spikes/min, respectively. These values were not significantly different and were similar to resting values (*P* > 0.05) before the first and after the last contractions of the protocol.

### Effect of contraction on MSNA

**Figure 3:**
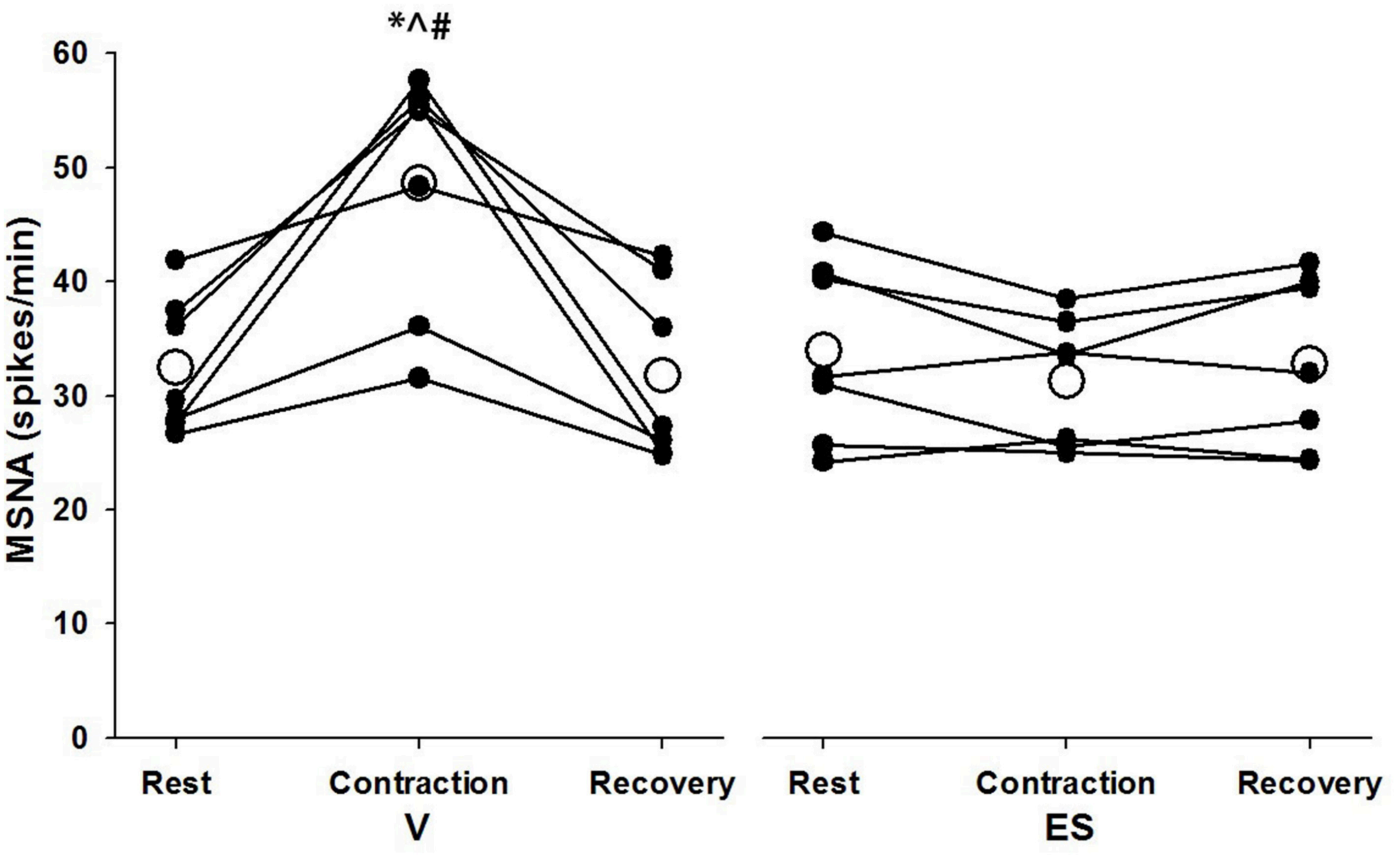
**MSNA spike frequency during 1-min periods of rest, contraction, and recovery for voluntary and electrically-evoked contractions**. Mean values are shown as open circles and individual values are shown as closed circles. ^*^Significant main effect of time (*P* < 0.01). **#**Significant main effect of condition (*P* = 0.05). ^∧^Significant interaction of time and condition (*P* < 0.01).

Successful recordings of MSNA were obtained during 48 voluntary contractions (mean = 6.9 ± 3.1 per subject) and 30 ES contractions (mean = 4.3 ± 1.5 per subject). The lower number for ES contractions was due to a higher incidence of failure to detect cardiac rhythmicity in the post-stimulus time histograms of negative-going spikes. For MSNA responses analyzed over 1-min periods before, during and after contractions, there was a significant effect of condition (*F* = 5.97, *P* = 0.05) and significant time-by-condition interaction (*F* = 21.5, *P* = 0.01) indicative of a greater increase in MSNA during V than ES (Figure 3). Compared with resting levels immediately before contraction, MSNA during V increased by 51 ± 34% (*P* < 0.01) whereas during ES the change in MSNA was small and not significant (−8 ± 12%, *P* = 0.11). MSNA during V was also greater (*P* < 0.01) than resting values immediately after contractions, whereas the same comparison for ES was not significant (*P* = 0.23).

**Figure 4:**
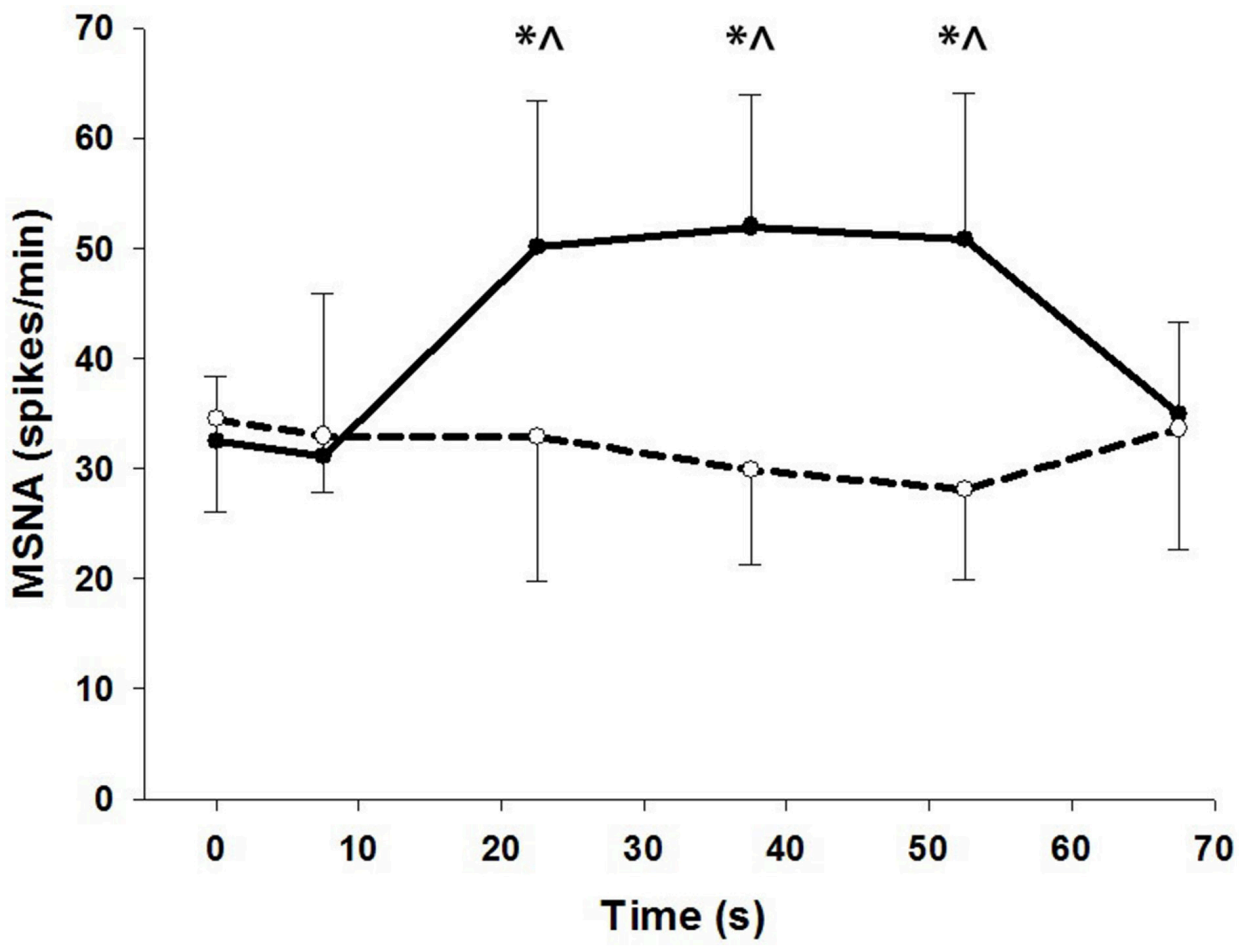
**MSNA at 15 s intervals during voluntary (closed circles) and electrically-evoked (open circles) contractions**. ^*^Significant main effect of condition (*P* < 0.03). ^∧^Significant interaction of time and condition (*P* < 0.01).

**Figure 5:**
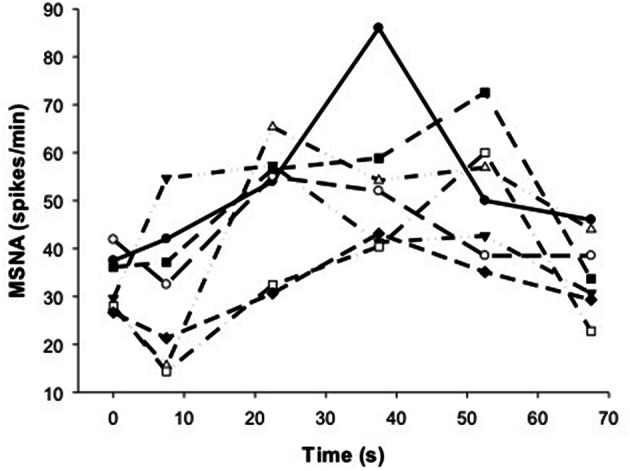
**Individual responses of MSNA before (*t* = 0 s), during (*t* = 7.5–52.5 s) and after (*t* = 67.5 s) voluntary contraction**.

To show the time-course of MSNA during contractions, MSNA was analyzed in 15 s intervals and compared with MSNA averaged over 1 min before and 15 s after the contractions (Figure 4). For voluntary contractions there was a significant main effect of time (one-way ANOVA: *F* = 7.17, *P* < 0.01): MSNA remained unchanged from resting levels during the initial 15 s of contraction (31.1 ± 14.9 spikes/min), increased by 54–65% during the remaining intervals of contraction (15–30 s = 50.2 ± 13.3 spikes/min; 30–45 s = 52.0 ± 12.0 spikes/min; 45–60 s = 50.8 ± 13.3 spikes/min), and then declined to levels after contraction (35.0 ± 8.3 spikes/min) which were not significantly different from levels before contraction. By contrast to voluntary contractions, MSNA during electrically-evoked contractions remained similar to levels before and after the contractions. To illustrate the variability in time-course of MSNA during the voluntary contractions, individual responses are shown in Figure 5.

To shed light on the validity of the spike frequency responses during V and ES, the effects of voluntary contraction on the burst frequency, amplitude and total activity of MSNA were assessed using a conventional technique and compared with estimates of spike frequency under the same condition. Over 60 s periods, the burst frequency, amplitude and total activity during rest vs. voluntary contraction were 18.4 ± 2.6 vs. 22.5 ± 2.2 bursts^.^min^−1^ (*P* < 0.05), 0.48 ± 0.16 vs. 0.71 ± 0.39 μV (*P* = 0.12) and 8.82 ± 3.24 vs. 15.62 ± 7.75 μV^.^min^−1^ (*P* < 0.05). The mean proportional changes in these values from rest to contraction were 22% (burst frequency), 48% (burst amplitude) and 77% (total MSNA). These proportional responses in MSNA measured using the conventional technique are similar to the 51% increase in spike frequency from rest to contraction (see above).

### Cardiovascular responses

Cardiovascular responses, EMG and force measured over 1-min intervals before, during and after contractions are shown in Table 1. Resting values of all variables before V and ES contractions were not significantly different. Although force was slightly higher during V than ES contractions (by ~2%MVC), this difference was not significant (*P* = 0.36). There was a small but significant main effect of time on MAP (*F* = 4.15, *P* = 0.04) during both types of contraction, with increases of 1–2 mmHg during V and ES compared with resting values. By contrast, heart rate was not significantly affected by either type of contraction. For both types of contraction, these cardiovascular responses were completed by the first 15-s interval of contraction (data not shown).

**Table 1:** **Changes in mean arterial pressure (MAP), heart rate (HR), EMG, and force during the 1 min volitionally generated (V) and electrically stimulated (ES) contractions**.

**Period**		**Rest**	**Contraction**	**Recovery**
SBP (mmHg)	V	141 ± 12	142 ± 13	141 ± 13
	ES	141 ± 11	143 ± 12	140 ± 11
DBP (mmHg)	V	65 ± 6	66 ± 6	65 ± 6
	ES	65 ± 6	65 ± 6	64 ± 7
MAP (mmHg)	V	85 ± 7	87 ± 7^*^	85 ± 7
	ES	85 ± 6	86 ± 7^*^	85 ± 7
HR (bpm)	V	61 ± 9	59 ± 9	62 ± 8
	ES	61 ± 9	60 ± 8	61 ± 10
EMG (% Max)	V	0 ± 0	9 ± 8^*^	0 ± 0
	ES	–	–	–
Force (% Max)	V	0 ± 0	7 ± 2^*^	0 ± 0
	ES	0 ± 0	5 ± 3^*^	0 ± 0

* *Significant main effect of time (P < 0.05). Mean ± SD*.

## Discussion

### Main findings

This study tested the hypothesis that a contraction-induced increase in MSNA to contracting muscle is greater during voluntary than electrically-evoked contractions. The present findings support this hypothesis and show that MSNA averaged over 1 min periods increased significantly during voluntary but not electrically-evoked contractions involving the same muscle. This effect appeared within 15–30 s of the onset of contraction and disappeared more rapidly after contraction. These findings implicate central command in the control of sympathetic nerve activity to contracting muscle and suggest that muscle afferent feedback is not involved, at least during the mild contractions studied here.

### Force and MSNA during voluntary contraction

Previously we showed that MSNA to active muscle increased in proportion to force during the first min of sustained, voluntary contractions (Boulton et al., 2014). Although this increasing effect of contraction on MSNA differed from earlier observations of the response of MSNA to contracting muscle, it is consistent with evidence of increased sympathetic outflow to contracting muscle obtained from measurements of noradrenaline spillover (see Introduction). The proportional effect of force on MSNA can be expressed as the percentage increase in MSNA above resting level normalized to the percentage increase in relative force (i.e., %MSNA/%MVC). In our previous study this effect of force on MSNA was similar at 10%MVC (9.0%MSNA/%MVC), 28%MVC (6.4%MSNA/%MVC), and 46%MVC (8.0%MSNA/%MVC) (Boulton et al., 2014). The present finding of a 51% increase in MSNA during 1-min voluntary contractions at 7%MVC equates to a proportional effect of ~7.7%MSNA/%MVC, which is consistent with previous observations and adds to the evidence of the potency of the MSNA response to contracting muscle. Moreover, this effect of force suggests that control of sympathetic outflow is linked to a mechanism involved in controlling and/or responsive to variations in muscle force.

#### Control mechanisms

Potential mechanisms involved in the control of MSNA to contracting muscle include descending neural drive from motor regions of the brain (i.e., central command) and afferent feedback from contracting muscle (i.e., mechanoreflex and metaboreflex). MSNA can also be influenced by the arterial baroreflex and pain, although in the present study neither of these are likely to explain the sustained increase in MSNA during voluntary contractions because mean arterial blood pressure increased by only 1–2 mmHg, heart rate did not change significantly, and contractions were not perceived to be painful.

#### Central command

Current perspectives suggest that the primary cardiovascular effect of central command during dynamic exercise is the resetting of the baroreflex and increase of heart rate through vagal withdrawal, whereas central command is thought to have minimal influence on muscle sympathetic outflow at most workloads (Rowell, 1993). Seminal work by Victor and colleagues showed that during sustained handgrip contractions (15–30%MVC) changes in central command induced by partial paralysis of muscle evoked only small increases in MSNA (Victor et al., 1989). The control of MSNA responses during dynamic exercise and static contractions is thought to be largely influenced by the muscle metaboreflex (Victor et al., 1987, 1989; Victor and Seals, 1989). However, this perspective of the role of central command in control of MSNA pertains mainly to evidence of MSNA measured in *inactive* limbs and does not address the influence of central command on MSNA to *active* skeletal muscle.

In the present study, voluntary contractions at a very low force (7%MVC) evoked relatively large increases in MSNA to active muscle, whereas electrically-evoked contractions failed to increase MSNA to this same muscle. Immediately after voluntary contractions, MSNA returned rapidly to resting levels and remained unchanged after electrical stimulation (Figures 4, 5). We showed previously that the effect of voluntary contraction on MSNA to active muscle is large and proportional to force (see above), not influenced by ischaemia, and declines rapidly after contraction even when ischaemia and metaboreflex activation persists (Boulton et al., 2014). Collectively, this evidence suggests that central command is involved directly in the control of MSNA to *active* muscle, which differs from the current perspective of MSNA and its control to *inactive* muscle.

Central command is clearly involved during voluntary contractions, but the lack of increase in heart rate and small increases in blood pressure observed in the present study might raise concern about the influence of central command on sympathetic outflow in the present experiment. Such small or negligible cardiovascular responses are typical of slightly more forceful voluntary and sustained contractions (15%MVC for 1 min) of anterior leg and foot muscles (Hansen et al., 1994). This suggests that variations in central command at lower forces of sustained contractions exert small or negligible effects on systemic cardiovascular responses. The present findings of a large increase in MSNA to active muscle during voluntary contractions relative to the heart rate and blood pressure responses suggests a much greater sensitivity of this local sympathetic response to central command and/or other factors compared with these systemic responses.

Despite evidence of involvement of central command during voluntary contractions, there was substantial delay in the rise in MSNA during voluntary contractions and which ranged between ~7.5–37.5 s for all subjects (Figure 5). This delay was surprising given evidence of the rapid effects of central command on other cardiorespiratory responses (Eldridge et al., 1981) and the rapid fall in MSNA after contraction (Figure 5). This asymmetry in timing between on- and off-responses might be influenced by the target force, since we observed a more rapid onset (within 15 s) of MSNA at higher compared with lower forces (46 vs. 10–28%MVC) but a similar and rapid decline in force after these contractions (Boulton et al., 2014). It is not clear if this effect of force on the timing of MSNA responses involves only central command or additional, faster-acting mechanisms at higher forces.

#### Muscle afferent feedback

Evidence of muscle mechanoreceptor and metaboreceptor involvement in MSNA responses is limited to *inactive* muscles. For example, mechanical stimulation induced by brief stretch applied to dorsiflexor muscles evoked a rapid (within 5 s) but transient increase in MSNA to the *contralateral* limb that was similar to the effect of moderate dorsiflexion contractions of the same duration (5 s) (Cui et al., 2006). Stimulation of mechanoreceptors would also be expected during contractions in the present study, but the failure of MSNA to increase during the first 15 s of both types of contractions and remaining 45 s of electrically-evoked contractions suggests that mechanically-mediated feedback did not contribute to the rise in MSNA during voluntary contractions. These findings also do not support involvement of metaboreceptor input. The metaboreflex, linked to muscle ischaemia, increases sympathetic outflow to *inactive* limbs during exercise (Rowell, 1993). Evidence of this includes the sustained increase in MSNA to inactive muscle following a contraction of a remote muscle which remains ischaemic by inflation of a limb cuff proximal to the contracted muscle (Hansen et al., 1994). We have shown that this effect is absent for MSNA to *active* muscle (Boulton et al., 2014), suggesting that chemically-mediated feedback from contracting muscle is not required for the control of MSNA to the same muscle.

#### Methodological considerations and limitations

Conventional measurement of MSNA is based on rectification and averaging (RMS) of the neurogram and assessment of frequency and/or amplitude of sympathetic “bursts” (Hansen et al., 1994; Boulton et al., 2014) or spectral analysis of this smoothed signal (Wallin et al., 1992; Boulton et al., 2014). These techniques could not be used to assess MSNA during electrically-evoked contractions because of the corrupting influence of electrical artifact. Therefore, we used an approach adopted in previous studies (Bent et al., 2006; Grewal et al., 2009; Hammam et al., 2011; Fatouleh and Macefield, 2013) which circumvented this problem and is based on detection and counting of negative spikes of sympathetic origin. These negative spikes constitute the elemental recording of sympathetic neuronal activity which underlie the more typical, RMS-transformed “burst” responses in most MSNA studies. The electrical artifact only obscured a small fraction of the recording period and at regular intervals (Figure 1A), which meant that negative, sympathetic spikes could be observed during most (>90%) of the contraction period and spike frequencies (per min) could be adjusted accordingly (see Methods). That the proportional effect of force of voluntary contraction on MSNA in this study was similar to our previous observations using RMS-transformed responses and spectral analysis of these responses supports our use of this alternative technique. Moreover, the similarity of proportional effects of *voluntary* contraction on spike frequency and conventional estimates of MSNA (Results) support the validity of using measurements of spike frequency to assess MSNA during electrical stimulation. Finally, the similarity of measurements of resting MSNA at the beginning and end of the experimental protocol, as well as between contractions, supports the stability of this measurement throughout the entire protocol and helped establish the clear effect of voluntary contraction on MSNA at such mild forces.

The differential effect of voluntary and electrically-evoked contractions on MSNA raises a question about the influence of difference in muscle contributions between these contractions. Electrical (intramuscular) stimulation involved only the *tibialis anterior* muscle, the most important dorsiflexor of the ankle. Although voluntary contractions involve this muscle supplemented by smaller contributions from toe extensors, subjects were asked not to extend the toes to maximize the contribution from tibialis anterior. Thus, it seems unlikely that any differences in activation of tibialis anterior and involvement of other muscles would be so large as to explain the different effects of the two types of contraction.

The present findings are limited to mild and sustained contractions. Contributions of central command and muscle feedback to control of sympathetic outflow to contracting muscle might differ at higher forces. However, use of mild contractions with minimal cardiovascular responses, baroreflex engagement and pain allowed us to better isolate mechanisms underlying the MSNA response and provide evidence of involvement of central command at low forces. The present findings pertain to sustained contractions and, although there is evidence of increased sympathetic outflow to contracting muscle during intermittent contractions (Savard et al., 1987), further studies of intermittent contractions are needed. More powerful experimental approaches are also required to establish the causal influence of central command on MSNA, such as those involving pharmacological manipulation of this influence while muscle force is maintained.

#### Perspective

Control of sympathetic outflow to *inactive muscle* is essentially important to the regulation of blood pressure; whereas control of sympathetic outflow to *active* muscle is critically important to controlling muscle vasodilation and hyperaemia during movement and exercise. Control of sympathetic neuronal activity (number and frequency) and noradrenaline release provides controlled constraint of the rapid and extensive vasodilation which occurs in contracting muscle. This constraint is required across a wide range of intensities (Savard et al., 1987; Sprangers et al., 1991; Masuki and Nose, 2003) and varied in proportion to intensity to help counterbalance the intensity-dependent change in vasodilator production. Present findings combined with recent work suggests that sympathetic outflow to contracting muscle is varied in proportion to the force of contraction and perhaps by central command. Given the extensive capacity for vasodilation in muscle, a centrally-mediated, feedforward mechanism of vascular constraint would constitute an important part of a hierarchical and complex system of vascular control in contracting muscle.

## Conclusion

This study confirms recent observations of robust increases in MSNA during mild voluntary contractions and, for the first time, shows that such a response is absent during electrically-evoked contractions of similar force. This differential effect implicates central command in the control of sympathetic outflow to contracting muscle and not muscle feedback, at least at mild forces. Further investigation of control of this sympathetic response at higher forces and during intermittent contractions or dynamic exercise is needed.

## Author contributions

Experiments were performed in the School of Medicine (University of Western Sydney). All authors were involved in the conception and design of the experiments, as well as the collection, analysis, and interpretation of data and writing or editing of this manuscript. All authors approved the final version of the manuscript.

### Conflict of interest statement

The authors declare that the research was conducted in the absence of any commercial or financial relationships that could be construed as a potential conflict of interest.

## References

[B1] Andersen P. Saltin B. (1985). Maximal perfusion of skeletal muscle in man. J. Physiol. 366, 233–249. 10.1113/jphysiol.1985.sp015794 4057091 PMC1193029

[B2] Bent L. R. Bolton P. S. Macefield V. G. (2006). Modulation of muscle sympathetic bursts by sinusoidal galvanic vestibular stimulation in human subjects. Exp. Brain Res. 174, 701–711. 10.1007/s00221-006-0515-6 16721608

[B3] Boulton D. Taylor C. E. Macefield V. G. Green S. (2014). Effect of contraction intensity on sympathetic nerve activity to active human skeletal muscle. Front. Physiol. 5:193. 10.3389/fphys.2014.00194 24917823 PMC4042086

[B4] Cui J. Blaha C. Moradkhan R. Gray K. S. Sinoway L. I. (2006). Muscle sympathetic nerve activity responses to dynamic passive muscle stretch in humans. J. Physiol. 576, 625–634. 10.1113/jphysiol.2006.116640 16873399 PMC1890351

[B5] Eldridge F. L. Millhorn D. E. Waldrop T. G. (1981). Exercise hyperpnea and locomotion: parallel activation from the hypothalamus. Science 211, 844–846. 10.1126/science.7466362 7466362

[B6] Fagius J. Wallin B. G. (1980). Sympathetic reflex latencies and conduction velocities in normal man. J. Neurol. Sci. 47, 433–448. 10.1016/0022-510X(80)90098-2 7420119

[B7] Fatouleh R. Macefield V. G. (2013). Cardiorespiratory coupling of sympathetic outflow in humans: a comparison of respiratory and cardiac modulation of sympathetic nerve activity to skin and muscle. Exp. Physiol. 98, 1327–1336. 10.1113/expphysiol.2013.072421 23625953

[B8] Green S. Thorp R. Reeder E. Donnelly J. Fordy G. (2011). Venous occlusion plethysmography versus Doppler ultrasound in the assessment of leg blood flow during calf exercise. Eur. J. Appl. Physiol. 111, 1889–1900. 10.1007/s00421-010-1819-6 21234593

[B9] Grewal T. James C. Macefield V. G. (2009). Frequency-dependent modulation of muscle sympathetic nerve activity by sinusoidal galvanic vestibular stimuation in human subjects. Exp. Brain Res. 197, 379–386. 10.1007/s00221-009-1926-y 19582437

[B10] Hammam E. James C. Dawood T. Macefield V. G. (2011). Low-frequency sinusoidal galvanic stimulation of the left and right vestibular nerves reveals two peaks of modulation in muscle sympathetic nerve activity. Exp. Brain Res. 213, 507–514. 10.1007/s00221-011-2800-2 21800255

[B11] Hansen J. Thomas G. D. Jacobsen T. N. Victor R. G. (1994). Muscle metaboreflex triggers parallel sympathetic activation in exercising and resting human skeletal muscle. Am. J. Physiol. 266, H2508–H2514. 8024012 10.1152/ajpheart.1994.266.6.H2508

[B12] Haug S. J. Segal S. S. (2005). Sympathetic neural inhibition of conducted vasodilatation along hamster feed arteries: complementary effects of a1- and a2-adrenoreceptor activation. J. Physiol. 563, 541–555. 10.1113/jphysiol.2004.072900 15576454 PMC1665587

[B13] Masuki S. Nose H. (2003). Arterial baroreflex control of muscle blood flow at the onset of voluntary locomotion in mice. J. Physiol. 553, 191–201. 10.1113/jphysiol.2003.047530 12937292 PMC2343480

[B14] Matsukawa K. (2012). Central command: control of cardiac sympathetic and vagal efferent nerve activity and the arterial baroreflex during spontaneous motor behaviour in animals. Exp. Physiol. 97, 20–28. 10.1113/expphysiol.2011.057661 21984731

[B15] Murphy M. N. Mizuno M. Mitchell J. H. Smith S. A. (2011). Cardiovascular regulation by skeletal muscle reflexes in health and disease. Am. J. Physiol. 301, H1191–H1204. 10.1152/ajpheart.00208.2011 21841019 PMC3197431

[B16] Reeder E. J. Green S. (2012). Dynamic response characteristics of muscle hyperaemia: effect of exercise intensity and relation to electromyographic activity. Eur. J. Appl. Physiol. 112, 3997–4013. 10.1007/s00421-012-2362-4 22441829

[B17] Rowell L. B. (1993). Human Cardiovascular Control. New York, NY: Oxford University Press.

[B18] Saltin B. (2007). Exercise hyperaemia: magnitude and aspects on regulation in humans. J. Physiol. 583, 819–823. 10.1113/jphysiol.2007.136309 17640931 PMC2277197

[B19] Saunders N. R. Pyke K. E. Tschakovsky M. E. (2005). Dynamic response characteristics of local muscle blood flow regulatory mechanisms in human forearm exercise. J. Appl. Physiol. 98, 1286–1296. 10.1152/japplphysiol.01118.2004 15579568

[B20] Savard G. Strange S. Kiens B. Richter E. A. Christensen N. J. Saltin B. (1987). Noradrenaline spillover during exercise in active versus resting skeletal muscle in man. Acta Physiol. Scand. 131, 507–515. 10.1111/j.1748-1716.1987.tb08270.x 3442240

[B21] Segal S. S. (2005). Regulation of blood flow in the microcirculation. Microcirculation 12, 33–45. 10.1080/10739680590895028 15804972

[B22] Sprangers R. L. H. Wesseling K. H. A. Imholtz L. T. Imholtz B. P. M. Wieling W. (1991). Initial blood pressure fall on stand up and exercise explained by changes in total peripheral resistance. J. Appl. Physiol. 70, 523–530. 2022542 10.1152/jappl.1991.70.2.523

[B23] Victor R. G. Pryor S. L. Secher N. H. Mitchell J. H. (1989). Effects of partial neuromuscular blockade on sympathetic nerve responses to static exercise in humans. Circ. Res. 65, 469–476. 2752552 10.1161/01.res.65.2.468

[B24] Victor R. G. Seals D. R. (1989). Reflex stimulation of sympathetic outflow during rhythmic exercise in humans. Am. J. Physiol. 257, H2017–H2024. 2603985 10.1152/ajpheart.1989.257.6.H2017

[B25] Victor R. G. Seals D. R. Mark A. L. (1987). Differential control of heart rate and sympathetic nerve activity during dynamic exercise. Insight from intraneural recordings in humans. J. Clin. Invest. 79, 508–516. 10.1172/JCI112841 3805279 PMC424115

[B26] Wallin B. G. Burke D. Gandevia S. C. (1992). Coherence between the sympathetic drives to relaxed and contracting muscles of different limbs of human subjects. J. Physiol. 455, 219–233. 10.1113/jphysiol.1992.sp019298 1484355 PMC1175641

